# Analysis of Johne’s disease ELISA status and associated performance parameters in Irish dairy cows

**DOI:** 10.1186/s12917-016-0667-y

**Published:** 2016-03-02

**Authors:** A. E. Kennedy, N. Byrne, A. B. Garcia, J. O’Mahony, R. G. Sayers

**Affiliations:** Animal & Bioscience Research Department, Animal & Grassland Research and Innovation Centre, Teagasc, Moorepark, Fermoy, Co. Cork Ireland; Department of Biological Sciences, Cork Institute of Technology, Bishopstown, Co. Cork Ireland

**Keywords:** Johne’s disease, ELISA, Dairy cow, Production

## Abstract

**Background:**

Infection with *Mycobacterium avium* subspecies *paratuberculosis* (MAP) has been associated with reductions in milk production in dairy cows and sub optimal fertility. The aim of this study was to highlight the production losses associated with testing MAP ELISA positive in Irish dairy cows. Secondary objectives included investigation of risk factors associated with testing MAP ELISA positive. A survey of management practices on study farms was also conducted, with examination of associations between management practices and herd MAP status.

Blood samples were collected from 4188 breeding animals on 22 farms. Samples were ELISA tested using the ID Screen Paratuberculosis Indirect Screening Test. Production parameters examined included milk yield, milk fat, milk protein, somatic cell count, and calving interval. The association between MAP ELISA status and production data was investigated using multi-level mixed models. Logistic regression was used to identify risk factors for testing JD blood ELISA positive at individual cow level and to identify associations between farm management practices and herd MAP status.

**Results:**

Data were available for 3528 cows. The apparent prevalence recorded was 7.4 %. Mixed model analysis revealed no statistically significant association between testing MAP ELISA positive and dairy cow production parameters. Risk factors associated with testing positive included larger sized herds being over twice more likely to test positive than smaller herds (OR 2.4 *P* = <0.001). Friesians were less likely to test positive relative to other breeds. A number of study farmers were engaged in management practices that have previously been identified as high risk for MAP transmission e.g., 73.1 % pooled colostrum and 84.6 % of study farmers used the calving area to house sick animals throughout the year. No significant associations however, were identified between farm management practices and herd MAP status.

**Conclusion:**

No production losses were identified; however an apparent prevalence of 7.4 % was recorded. With the abolition of EU milk quotas herd size in Ireland is expanding, as herds included in this study were larger than the national average, results may be indicative of future JD levels if no JD control programmes are implemented to minimise transmission.

## Background

Clinical and sub-clinical manifestations of disease can result in reductions in animal productivity leading to reduced farm profits [1, 2]. Cost-benefit analyses are often conducted to highlight these economic losses in order to promote the use of disease control schemes [3]. Johne’s disease (JD) is a chronic granulomatous enteritis of ruminants and is caused by the bacterium *Mycobacterium avium* subspecies *paratuberculosis* (MAP) [4]. Infection with MAP has been associated with production losses at farm level although equivocal results are reported [5]. Due to a prolonged subclinical phase, variable disease progression and immune response [6], diagnosis of MAP infection is challenging. Enzyme linked immunosorbant assay (ELISA), is a popular method of testing for MAP due to its speed and low cost [7]. Despite variable sensitivity and specificity [8, 9], ELISA testing is often the method of choice for epidemiological studies and herd-based diagnosis [10], and forms the basis of a number of international control programmes [11]. It is also a common diagnostic tool used in economic studies of Johne’s [5].

Economic losses reported due to infection with MAP include decreased slaughter value [12], reductions in milk production in dairy cows [2, 13], sub-optimal fertility [14], and an increase in cow replacement costs [15]. Although losses in clinically affected animals are well defined [15, 16], losses due to subclinical infection appear less well characterised [5]. An association between subclinical MAP infection and decreased milk yield (MY) has been identified in a number of studies [17–19]. In contrast a number of additional studies have identified no such association [20–22]. Similarly, conflicting reports exist regarding an association between subclinical MAP infection and milk fat (MF) or milk protein (MP) content [17, 21, 23]. An increased interval from calving to conception in ELISA positive cows has also been reported [14] in contrast to a different study showing ELISA positive cows to have fewer non-pregnant days [23]. Similarly, JE Lombard, FB Garry, BJ McCluskey and BA Wagner [23] recorded no association between MAP ELISA positivity and somatic cell count (SCC) which again conflicts with other studies [20, 24] that reported an associated increase in SCC in MAP ELISA positive dairy cows.

The variability that exists across diagnostic test methods may, in part, explain the conflicting performance-related data reported across various studies [5, 25]. Geographical location, choice of sample matrix, size of study population, cow breeds, and positive cow classification also differs across studies. Serum samples were used for diagnostic purposes in some studies [22, 23], while others used individual milk samples [19]. MG Gonda, YM Chang, GE Shook, MT Collins and BW Kirkpatrick [18] defined a JD positive cow on the basis of serum ELISA and/or faecal culture results. Study sample sizes ranged from less than 1000 [22] to 35,591 dairy cows [19] with other studies only examining a single cow breed [18, 22]. In this regard, it is important that data continue to be generated on similar cow populations, using similar study designs to improve the degree of confidence that exists in the likely impact on production in MAP positive dairy cows.

The prevalence of JD is believed to be increasing in the Republic of Ireland over the last 10 years [26]. In order to prevent further increases in MAP infection, improvements in control are required on Irish dairy farms. Although a previous Irish study [22] identified no significant effect of MAP sero-status on herd performance in 2004–2005, the increasing prevalence of MAP may now be impacting on Irish dairy production. The dairy landscape in Europe is changing due to the abolition of EU milk quotas in 2015 [27] and Irish farmers have been expanding herds over the past number of years [28]. Given Ireland’s increasing herd size, a known risk factor for testing MAP positive [29], this study aimed to investigate the current impact of MAP ELISA sero-positivity on individual cow milk production, SCC and calving interval. Secondary objectives included investigation of risk factors (e.g., breed, parity, calf and calving management) associated with testing MAP ELISA positive in Irish dairy herds and investigating the strength of correlation between milk and serum ELISA results.

## Methods

### Study population and sampling

**Fig. 1:**
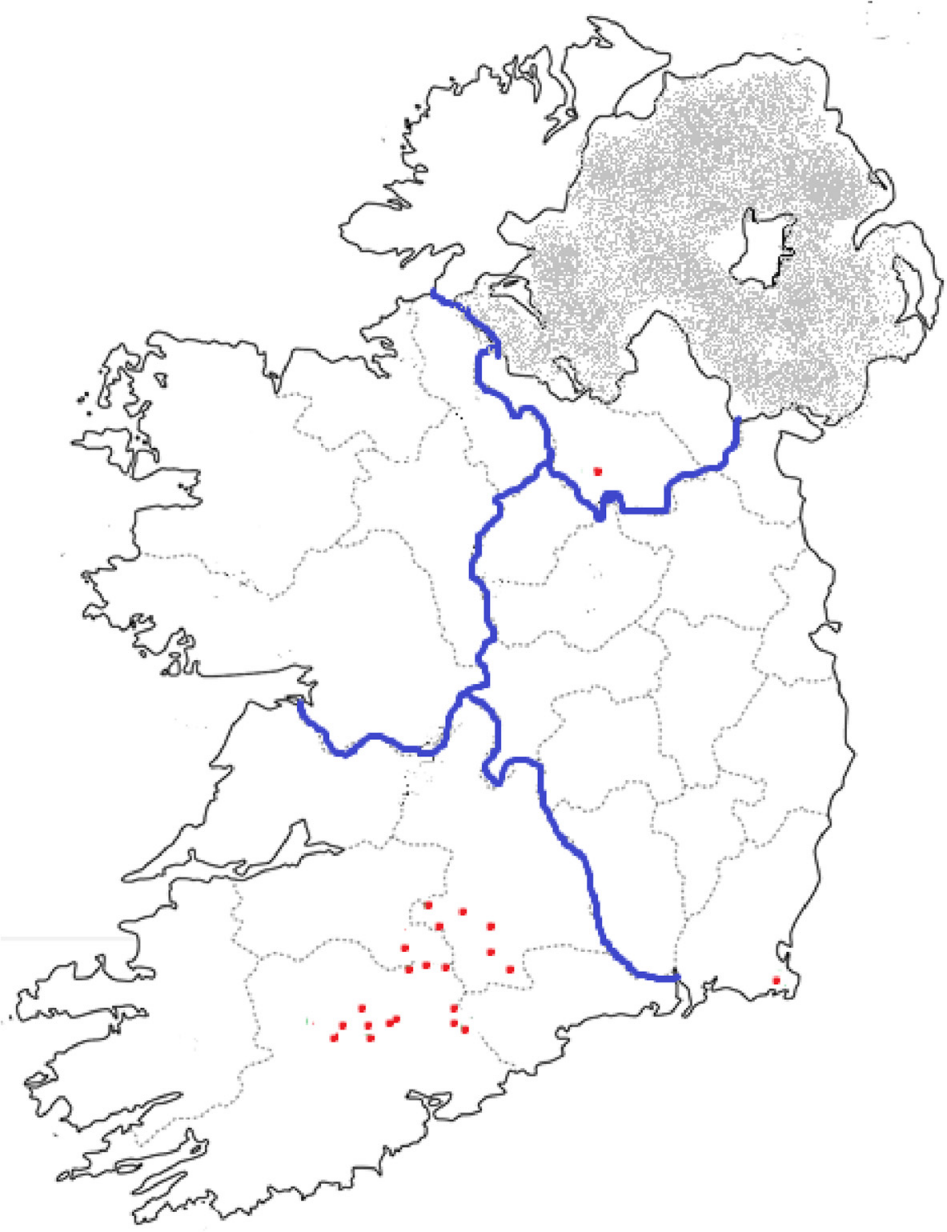
Map showing location of study farms. The majority of study farms were located in the dairy dense province of Munster, with one farm located in Leinster and Ulster

Blood samples were collected from all breeding animals aged over 2 years on 22 Irish dairy farms in 2012. This was conducted under licence from the Department of Health and Children, the licencing authority in Ireland at the time of the study. The location of study herds is included in Fig. 1. All but two herds were located in the dairy dense province of Munster, Ireland (south-western region) with an additional herd in each of Ulster and Leinster. A milk sample was also collected from each cow blood sampled on 17 of these farms. All study animals were observed by a veterinary surgeon during sampling visits and none displayed overt clinical signs of JD.

### Sample testing

Serum and milk samples were tested by a commercial ISO accredited laboratory (Enfer Labs, Kildare, Ireland) using the ID Screen Paratuberculosis Indirect Screening Test (ID Vet, Montpellier, France). This ELISA has a reported sensitivity (Se) of 41.5 % and specificity (Sp) of 99.42 [30]. The test is an *M. phlei* absorbed ELISA detecting anti-MAP immunoglobulin G (IgG). This ELISA was chosen as it is approved for use in Ireland’s national voluntary JD pilot control programme^1^ and displayed the highest overall accuracy of four commercial ELISA kits investigated by ROC analysis [30]. Results were reported as sample to positive ratio (S/P ratio) calculated using the formula S/P ratio = ((OD _Sample_ – OD _Positive control_) ÷ (OD _Positive control_ – OD _Negative control_) × 100). Animals were assigned MAP status (positive or negative) according to kit manufacturer interpretation (‘kit-interpretation’), with serum results of S/P ≥ 70 % classified as positive. A more severe test interpretation was also applied to blood ELISA results only in order to achieve increased test sensitivity, similar to the revised ELISA cut-off used by MT Collins, SJ Wells, KR Petrini, JE Collins, RD Schultz and RH Whitlock [31]. In this current study, instead of arbitrarily choosing a more sensitive positive cut-off, the mean S/P ratio of cows classified as negative using kit interpretation plus three times the standard deviation was used. This yielded a new ‘severe interpretation’ positive cut off of S/P ≥51.59.

### Individual cow performance data

Production data for each individual cow were downloaded from the Irish Cattle Breeding Federation (ICBF) database. This database holds production data on milk recording herds to support breeding decisions and enhance genetic gain in Irish dairy herds. Production parameters downloaded included 305 day [32] MY, MF, MP, SCC, and calving interval (CI). Only calving intervals of greater than 250 and less than 500 days were retained for analysis to allow comparison with K Hoogendam, E Richardson and JF Mee [22]. Additional cow related data downloaded from the ICBF database included parity, breed, and economic breeding index (EBI; a profit index measured in Euro, aimed at identifying the most profitable cows for breeding dairy herd replacements [33]).

### Survey data

Data relating to farm management practices on study farms were collected during scheduled farm visits. Questions focused on management practices which have previously been identified in the literature as being associated with a high risk of MAP transmission.

### Dataset construction

The production performance for each cow was matched to her ELISA result to create the dataset of statistical analysis. Both milk and blood ELISA results were included where available and the ‘kit-interpretation’ used to classify cows as positive and negative. A further dataset was constructed in an effort to identify production losses experienced by cows recording ELISA results near the manufacturers’ cut off point. This dataset was constructed using the ‘severe-interpretation’ ELISA positive cut-off. Both blood data sets were used for the mixed model analysis with the only ‘kit-interpretation’ dataset used for prevalence investigations and investigating the correlation between blood and milk ELISA results. To investigate the correlation between paired milk and serum ELISA results, Spearman correlation (*rs*) was performed on categorical results i.e., cows classified as positive or negative.

### Data analysis

Data manipulation and graphical representations including box plots were completed in Excel (MS 2010). Generalised linear latent and mixed models (gllamm), logistic regression, and Spearman correlations were performed using Stata (Version 12).

### Prevalence calculation

The apparent prevalence’s (Ap) at animal level and within herd level were calculated as total number of test positive animals out of the total number of animals tested. To estimate the true prevalence (Tp) of positive animals, a Bayesian approach and Gibbs sampling method was applied using an online epidemiological calculator (Epitools) [34]. This calculator requires prior estimates of the true prevalence and test sensitivity and specificity, based on previous data or expert knowledge. These estimates are made as beta probability distributions, with parameters alpha and beta. Alpha and beta can be calculated provided estimates of the mode and 5 or 95 % confidence limits are available from expert opinion. When the mode was between 0.5 and 1, the 95th percentile was entered into the Beta distribution utilities calculator, and the 5th percentile entered if between 0.5 and 1. Prior estimates for true prevalence were based on a national survey which reported a prevalence of 3.3 % from a study population of 15,558 animals aged over 2 years [26]. Beta probability distributions for ELISA Se and Sp were compiled based on estimates from peer-reviewed literature [30, 31], expert opinion from Irish veterinary practitioners, veterinary officers, Teagasc veterinary and agricultural researchers, and a comprehensive longitudinal farm study involving ELISA screening and confirmatory testing using faecal culture and post-mortem examination. The estimates and beta distributions used to determine Tp are outlined in Table 1. Upper and lower confidence limits for Tp calculation were set at 97.5 and 2.5 % respectively. Outputs reported in this study are median values of posterior distributions for prevalence, sensitivity and specificity.

**Table 1:** Estimates for use in true prevalence calculation

	Estimates of posterior distributions	Beta distribution	
		Alpha	Beta
Animal Prevalence	0.033 (0.072)^a^	5.2021	124.1336
Sensitivity	0.41 (0.587)^a^	9.9689	13.5179
Specificity	0.99 (0.47)^a^	4.0322	1.0306

The online epidemiological calculator (Epitools) used to calculate true prevalence requires prior estimates of the true prevalence and test sensitivity and specificity, based on previous data or expert knowledge. These estimates are made as Beta probability distributions, with parameters alpha and beta. Alpha and beta can be calculated provided estimates of the mode and 5 or 95 % confidence limits are available from expert opinion

Initial values represent the mode, with the value in brackets representing either the 5^th^ or 95th percentile

^a^When the estimated value was between 0 and 0.5 the 95th percentile was chosen, and when the estimate was between 0.5 and 1 the 5th percentile was chosen

### Associations between cow performance and MAP ELISA status

All data were visually assessed for normality using ladder of powers histograms in Stata. The association between MAP ELISA status and production data was investigated using gllamm. Models accounted for random effects of cow nested within herd. Covariates were retained in the final multivariable models on the basis of the highest reduction in the Bayesian information criterion (BIC). Covariates examined in each model were breed (Friesian/Friesian crosses (FRx), Jersey/Jersey crosses (JEx),. Norwegian Red/Norwegian Red crosses (Redx), other), parity (1, 2, 3, 4, 5, 6, 7, 8, 9, ≥10), EBI (categorised into quartiles, category 1 being the highest quartile and category 4 the lowest) and herd size (≤150 cows, >150 cows). This number was chosen as average herd size of study herds was 153 cows. Second-level interactions between covariates were also examined. Values of *P* < 0.05 were considered significant.

### Associations between breed, herd size, parity, EBI and MAP ELISA status

Logistic regression was used to identify risk factors for testing MAP blood ELISA positive at individual cow level (dependent variable). Independent variables examined in regression included breed, herd size, parity and EBI. These variables were coded as described for gllamm models. Herd of origin was forced into all models. A manual backwards elimination with a forward step was performed for each model. Interactions between variables were also examined. Variables recording a significance level of *P* < 0.05 were retained in the model and are reported.

### Associations between farm management practices and herd MAP status

Logistic regression was used to identify associations between testing MAP blood ELISA positive at a herd level and farm management practices (survey responses). A herd was classified as positive if a minimum of one blood ELISA positive result was identified. Herd size was included in the model as a covariate, as larger herds are more likely to test positive [29]. Again a manual backwards elimination was performed with interactions between variables examined. Variables recording *P* < 0.05 were considered significant.

## Results

### Descriptive data

Samples were collected from 4188 breeding animals. Production data were available for 3528 dairy cows, the remainder (*n* = 660) being excluded from further analysis. These exclusions consisted of misidentified animals, breeding bulls, and beef cows in herds having mixed beef and dairy enterprises. Of the 22 herds, nine contained >150 cows.

**Fig. 2:**
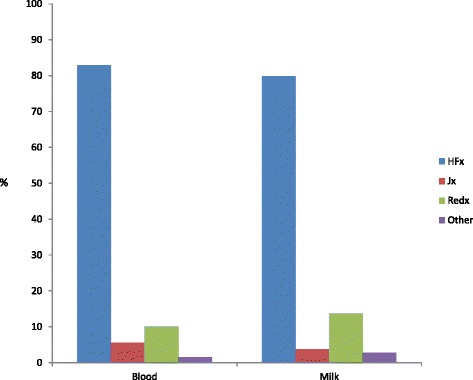
Proportion of animals tested belonging to each breed. The predominant breed tested using both milk and blood ELISA was Friesian. HFx: Friesian, JX: Jersey, Red: Norwegian red

**Fig. 3:**
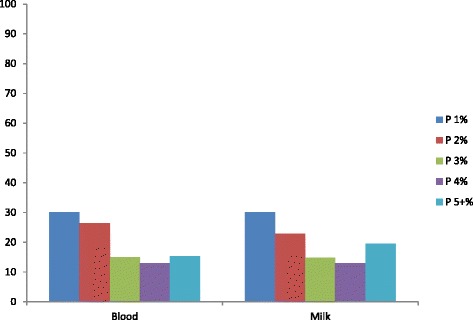
Proportion of animals tested belonging to each parity. The majority of animals tested were of parity 1 or parity 2. P = parity

The predominant breed sampled was FRx (82.7 %), the remaining 10.3, 5.5 and 1.5 % being Redx, Jersey, and other, respectively (Fig. 2). The majority of animals tested were parity1 (30.1 %) or parity 2 (26.4 %) (Fig. 3).

### Prevalence

**Fig. 4:**
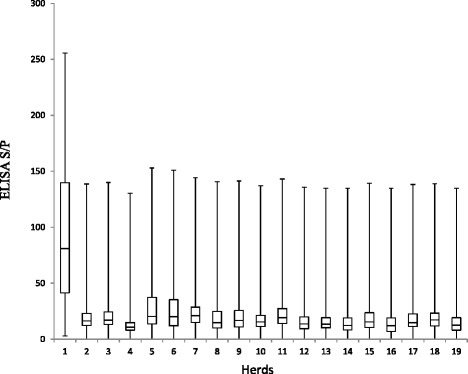
Box plot showing range of S/P ratios across all herds that recorded at least one positive animal. Over half of the animals in herd 1 tested positive

**Fig. 5:**
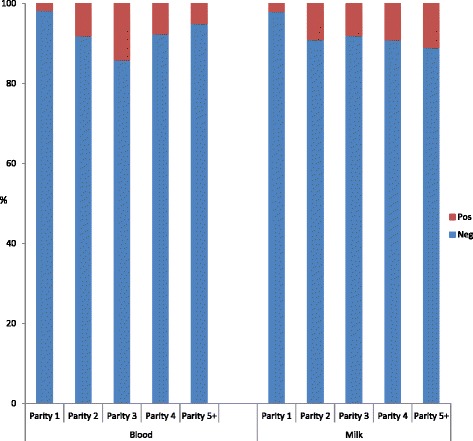
Proportion of animals testing positive per parity. The highest proportions of animals testing blood ELISA positive were of parity 3

**Fig. 6:**
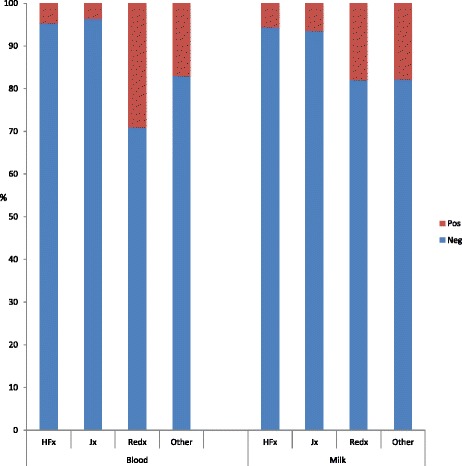
Proportion of positive results recorded across each breed. Norwegian reds were proportionally the predominant breed testing positive. The majority of this breed testing positive however originated from the same herd. HFx: Friesian, JX: Jersey, Red: Norwegian red

The highest within herd Ap recorded on any single farm was 56 %. Box plots showing range of S/P ratios across all herds with at least one positive animal are shown in Fig. 4. The overall study Ap recorded utilising blood MAP ELISA was 7.4 %. Based on the Bayesian analysis, Tp was estimated at 3.8 %, with a median output test Se and Sp of 41.6 and 94 %, respectively. All cows tested in three herds recorded negative blood MAP ELISA results and these herds contained <150 cows. A breakdown of the positives across parity, breed, and herd size are outlined in Table 2. The highest proportion of animals testing ELISA positive were third parity and Redx being proportionally the predominant breed testing positive (Figs. 5 and 6, respectively), although the majority of Redx testing positive were from a single herd. Many of the animals testing positive in this herd were born in the same year.

**Table 2:** Breakdown of positives across parity and breed

Matrix	Total tested	P 1 %	P 2 %	P 3 %	P 4 %	P 5 %	HFX %	Jx %	Redx %	Other %
Blood	3528	30.1	26.4	14.9	13	15.6	82.7	5.5	10.3	1.5
Milk	1686	30	22.8	14.7	13	19.5	80.5	3.7	14.1	1.7
	Total Positive(n)	P 1 %	P 2 %	P 3 %	P 4 %	P 5 %	HFX%	Jx%	Redx%	Other%
Blood	261	29.5	28.7	14.2	10.0	17.6	53.6	2.4	39.8	4.2
Milk	131	27.5	16.8	13.7	13	29	56.4	3.9	32.8	6.9

*P* parity

### Milk ELISA results

**Fig. 7:**
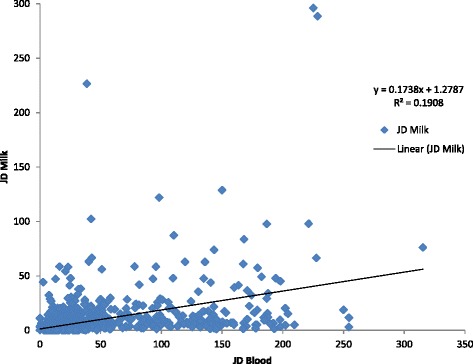
Scatter plot showing the relationship between matched blood and milk samples. An R^2^ value of 0.1908 was obtained

A total of 131 from 1696 cows available for analysis, tested milk ELISA positive for MAP. Of these, 61 also tested positive on blood. The remaining 70 animals recording positive milk ELISA results tested negative on blood ELISA. It should be noted that samples from 47 ‘milk positive blood negative’ cows were collected in September/October/November. These samples would therefore have been collected during late lactation in the Irish Spring calving dairy system. Further to this a number of the blood results, although classified as negative, were approaching the manufacturer cut off point of 70 S/P, possibly explaining the discrepancy between milk and blood ELISA. Spearman correlation yielded a ρ value of 0.19 indicating poor correlation between milk and blood test results at a categorical level (Fig. 7).

### Management practices- survey

**Fig. 8:**
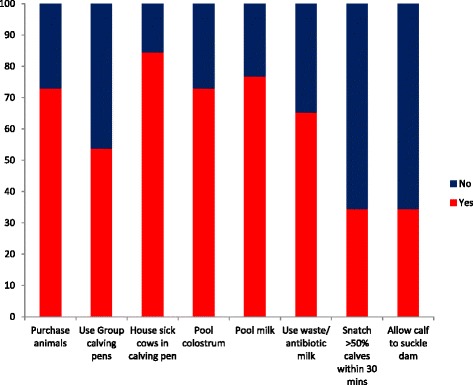
Responses to survey questions. The questions focus on management practices that have previously been associated with JD transmission. The high risk practices for JD transmission are shown in red

The majority (73.1 %) of respondents purchased animals onto their farms. Approximately, three quarters of study farmers fed calves pooled colostrum (73.1 %) and pooled milk (76.9 %). Additionally, milk not fit for sale i.e., milk containing antibiotic residues or milk from sick/mastitic cows was used to feed calves on 65.2 % of study farms. Group calving pens were used by 54 % of study farmers. The majority (84.6 %) of study farmers also used the calving area to house sick animals throughout the year. A high proportion of farmers removed calves from the calving area within 30 min of birth (65.4 %). Similarly, the majority of study farmers didn’t allow calves to suckle the dam (65.4 %) (Fig. 8).

### Production data

Mean milk yield was 5494 kgs. Mean milk fat and protein was 240 kgs and 196 kgs respectively. As a percentage of yields this equated to 4.4 % milk fat and 3.6 % protein.

### Mixed model analysis of production parameters

Multilevel mixed model analysis revealed no statistically significant association between testing MAP ELISA positive and MY, milk solids, CI, and SCC. Similarly, analysis of the ‘severe interpretation’ dataset again revealed no statistically significant differences (Table 3). No statistically significant association between testing milk ELISA positive and MY, milk solids, CI, and SCC was identified.

**Table 3:** Results from multilevel mixed model analysis

Dataset Name	Coefficient: JD positive vs. JD negative	Stnd Error	*P* Value
Dependent Variable			
Blood			
Milk Kgs	−8.7	47.3	0.854
Protein %	0.14	0.01	0.424
Fat %	−0.03	0.03	0.343
Calving Interval	−3.2	2.0	0.098
SCC	10.3	21.7	0.635
Severe Interpretation			
Milk Kgs	−3.91	52.5	0.9406
Protein %	−0.01	0.01	0.6745
Fat %	−0.02	0.03	0.5257
Calving Interval	−4.52	5.04	0.3695
SCC	10.4	22.4	0.6432

No significant associations between production parameters and sero status were identified utilising the manufacture cut of point of 70 or the severe interpretation cut off point of 51.59

*P* Value: Significant *P* <0.05

### Associations between breed, herd size, parity, EBI and MAP ELISA status

Statistically significant results of logistic regression analysis are included in Table 4. Larger herds were over twice more likely to test positive than smaller herds. Redx and breeds classed as other were more likely to test JD ELISA positive than FRx (OR 6.5, 5.5 respectively). No significant associations were highlighted between parity, EBI and MAP ELISA status.

**Table 4:** Logistic regression- Significant associations between testing MAP ELISA positive and independent variables

Dependent Variable	Odds Ratio	*P* Value	Conf. Interval (95 %)	Model^a^
Independent Variable				
Johne’s disease ELISA positive				
Herd size				Herd of origin Breed Herd size Parity EBI
Herd size >150 cows vs. herd size < 150 cows	2.4	<0.001	1.7, 3.4	
Breeds				
Red x vs. FRx	6.5	<0.001	4.8, 8.9	
Other vs. FRx	5.5	<0.001	2.5, 12.2	
Red x vs. Jex	12.2	<0.001	5.2, 28.6	
Other vs. Jex	10.3	<0.001	3.3, 32.1	

Larger sized herds were more likely to test positive compared to smaller sized herds. Friesians were less likely to test positive relative to other breeds examined

*P* Value: Significant *P* <0.05. Only significant results shown ^a^Outlines the independent variables included in the logistic regression model

### Associations between farm management practices and herd MAP status

No significant association was identified between calving area, calf feeding management practices and MAP ELISA status.

## Discussion

Economic losses are often reported due to JD [35]. Given Ireland’s increasing herd size, a known risk factor for testing MAP positive [29], this study aimed to investigate the current impact of MAP ELISA sero-positivity on individual cow performance. Secondary objectives included investigation of risk factors (e.g., breed, parity, calf and calving management) associated with testing MAP ELISA positive in Irish dairy herds and investigating the strength of correlation between milk and serum ELISA results.

Two previous Irish reports have highlighted significant losses in JD clinically affected animals [13, 16], but are limited to individual farm case studies as opposed to across farm studies. An additional Irish study involving 34 herds, however, reported no statistical effect of JD sero-status (sub-clinical cows) on Irish dairy cow milk and fertility performance parameters [22]. As the study conducted by K Hoogendam, E Richardson and JF Mee [22]. involved a sample size of 949 and only 11 serologically positive individuals, it was necessary to conduct a larger study in Irish dairy herds. Both the overall sample size and number of ELISA positive animals detected, were considerably higher in the current study (3528 cows and 261 positives) which would greatly increase the confidence in the findings reported. It was unexpected, therefore, that a continuing lack of statistically significant association between JD sero-positivity and performance in Irish dairy cows was highlighted. This is not particular to Ireland; with additional international studies reporting similar findings [5, 21]. It may be suggested, therefore, that use of poor sensitivity, and possibly specificity, ELISA tests as are currently available is not an ideal study design in order to detect sub-clinical losses due to JD infection. Studies, however, have detected losses in sero-positive individuals [19, 23] and as ELISA testing forms an integral part of many international control programmes [11, 36], studies aimed at highlighting production losses associated with JD ELISA status are important additions to the global JD database. Further such studies may allow an analysis of geographical differences in the impact of sub-clinical JD across various countries, which in turn could assist in identifying protective or stimulatory factors with regard to JD infection.

The impact on production associated with testing MAP ELISA positive will differ depending on choice of diagnostic test. This is because the accuracy of MAP testing differs across both kits and stage of infection in the animal tested [5]. Indeed the poor correlation between the use of milk and blood samples identified in the current study serves to clearly highlight the considerable variability between diagnostic test methods, especially at different stages in lactation as highlighted in this study and others [37, 38]. Indeed as the specificity of MAP ELISA is not 100 % [9], it is possible that a proportion of the animals identified as positive in the current study may be false positives. The Bayesian estimates of Tp would support this in that it indicates a lower level of true infection than the apparent prevalence recorded. Identification of false positives and false negatives due to less than optimal test sensitivity and specificity would lead to mis-categorisation of individuals hence reducing the likelihood of detecting association between performance and ELISA status. This again stresses the importance of using large sample size studies for these analyses. The current study strove to correct for poor test sensitivity by using a ‘severe-interpretation’ yet production differences remained unidentifiable.

A number of environmental mycobacteria have been identified in Ireland [39]. Environmental mycobacteria are known to contribute to false positive ELISA results [40], potentially allowing interference with results in the current study. As faecal culture positive cows have been shown to have consistently larger effects on all production traits compared to MAP ELISA positive cows [18], a limit of this study is the lack of faecal culture. A study conducted in the same region as the current study however, showed moderate agreement between ELISA testing and culture techniques [41], indicating there is a level of agreement between ELISA and culture tests in the region. As, however, results from this study indicate no economic effect of testing ELISA positive, it may indicate that future economic investigations in Ireland should not be conducted utilising ELISA alone. It may also prove worthwhile in future studies to increase the ELISA test kit cut-off as a means of potentially improving test specificity.

It was important to identify whether farmers engaged in good Johne’s management in this study to examine the influence of the farmer as a protective influence. Not only were farmers in the current study not engaging in protective practices, a higher level of adoption of certain high risk management practices than previously reported in Ireland was recorded. The current study reported 65 % of study farmers feeding waste/antibiotic milk to calves compared to 59.6 % in the previous national study [42]. Use of the calving area to house sick animals was also reported at a much higher level than previous national [42] and international studies [43]. Results from this study also show higher usage of group calving pens than that reported elsewhere [41, 43] possibly placing study farms at increased risk of the incidence of diarrhoea, including salmonella and JD [44, 45]. Indeed maintenance of a closed herd is a key element of general herd biosecurity [46] however over 70 % of study farmer’s reported not operating a closed herd. Given the high level of high risk management practices reported in the current study and a previous study [42] it is important to provide information to farmers about the pathogenesis and transmission of JD to minimise future JD levels. As almost all participants were utilising high risk management practices, it is perhaps unsurprising that no significant differences were identified between management practices adopted on test positive and test negative herds.

Although a high number of MAP ELISA positive animals were identified, no animal was observed to be displaying clinical signs of JD at the time of sampling. As typical results in relation to JD risk factors were identified i.e., larger herds being more likely to test positive, perhaps it indicates an unidentified element exists within Irish dairying systems that limits the production effects and clinical signs on Irish cattle. Potential protective effects may include the widespread use of grass based systems in Ireland or the lower average herd size compared to other countries [47, 48]. It may also however relate to the level of environmental mycobacteria present and the extensive TB testing programme that operates in Ireland. Infection with MAP can lead to false positive reactions on the intradermal skin test for bTB [49]. As a bTB test is administered on at least a single occasion annually to every bovine in Ireland, removal of ‘MAP reactors’ prior to the development of clinical signs is possible, potentially explaining negligible losses.

Further to this, although the annual culling rate of approximately 21 % is lower than some countries [50], this level of culling may have led to the removal of some MAP infected animals prior to the onset of clinical signs. Currently the dairying sector in Ireland and the EU in general is entering a period of change due to the abolition of EU milk quota restrictions. The Irish dairy sector has set targets to increase dairy output by 50 % by 2020 (Food Harvest 2020). In order to meet this 2020 target, there is a requirement to increase cow numbers by 350,000 in Ireland by 2020, compared to average cow numbers between 2007 and 2009 [51]. With the abolition of EU milk quotas and the intent of many farms to expand in size [27], it may be that animals that would previously have been culled may in future be retained in the herd, potentially leading to an increased risk of MAP transmission. As this study involves herds larger than the national average herd size and results show an increased number of animals testing positive than previous Irish studies, it will be important to monitor the production effects associated with testing JD positive, as national herd size expands.

The greater likelihood of Redx to test positive relative to JEx and HFx was a surprising result from this study as there is a low level of JD in the breed’s country of origin and also due to a speculated increased resistance to JD in Norwegian red cattle [52]. Norwegian Reds are commonly utilised in breeding programmes to increase herd genetic merit [53]. In the 1990’s a large number of dairy cattle were imported to Ireland from continental Europe [16]. The practice of importing cattle from abroad has been associated with increased risk of testing JD positive [29]. It is possible that farms in the present study that utilised Norwegian Reds to improve herd genetic merit may have previously imported cows from abroad, possibly allowing the establishment of JD within the herd, facilitating current transmission to Norwegian Red cows. Indeed it is possible that herds interested in cross breeding and improving herd genetic merit may be more progressive and potentially larger in size further exacerbating the increased odds of testing positive.

## Conclusion

An Ap and Tp of 7.4 and 3.8 %, respectively was recorded in this study and no statistically significant production losses were identified although the majority of study farms engaged in high risk management practices for JD transmission. Although the average dairy herd size in Ireland is relatively small, which may be protective against MAP transmission, the abolition of EU milk quotas is leading to dramatic increases in herd size in Ireland. As this study reiterates the increased risk of JD in larger herds it will be necessary to repeat economic studies to monitor the impact of changing demographics in national herds.

## Abbreviations

Ap/Tp: Apparent/True Prevalence
CI: calving interval
JD: Johne’s disease
MAP: *Mycobacterium avium* subspecies *paratuberculosis*
MY/MF/MP: milk yield/fat/protein
Se: sensitivity
SCC: somatic cell count
Sp: specificity

## Footnotes

1 www.animalhealthireland.ie
